# Lignin composition is more important than content for maize stem cell wall degradation

**DOI:** 10.1002/jsfa.8630

**Published:** 2017-09-25

**Authors:** Yuan He, Thibaut MB Mouthier, Mirjam A Kabel, Jan Dijkstra, Wouter H Hendriks, Paul C Struik, John W Cone

**Affiliations:** ^1^ Animal Nutrition Group Wageningen University & Research, 6700 AH Wageningen the Netherlands; ^2^ Food Chemistry Wageningen University & Research, 6700 AA Wageningen the Netherlands; ^3^ Centre for Crop Systems Analysis Wageningen University & Research, 6700 AK Wageningen the Netherlands

**Keywords:** lignin content, lignin composition, cell wall degradation, maize stem, gas production, in vitro fermentation

## Abstract

**BACKGROUND:**

The relationship between the chemical and molecular properties – in particular the (acid detergent) lignin (ADL) content and composition expressed as the ratio between syringyl and guaiacyl compounds (S:G ratio) – of maize stems and in vitro gas production was studied in order to determine which is more important in the degradability of maize stem cell walls in the rumen of ruminants. Different internodes from two contrasting maize cultivars (Ambrosini and Aastar) were harvested during the growing season.

**RESULTS:**

The ADL content decreased with greater internode number within the stem, whereas the ADL content fluctuated during the season for both cultivars. The S:G ratio was lower in younger tissue (greater internode number or earlier harvest date) in both cultivars. For the gas produced between 3 and 20 h, representing the fermentation of cell walls in rumen fluid, a stronger correlation (R^2^ = 0.80) was found with the S:G ratio than with the ADL content (R^2^ = 0.68). The relationship between ADL content or S:G ratio and 72‐h gas production, representing total organic matter degradation, was weaker than that with gas produced between 3 and 20 h.

**CONCLUSION:**

The S:G ratio plays a more dominant role than ADL content in maize stem cell wall degradation. © 2017 The Authors. *Journal of The Science of Food and Agriculture* published by John Wiley & Sons Ltd on behalf of Society of Chemical Industry.

## INTRODUCTION

Forage maize is an important forage for high‐yielding dairy cows in most areas of the world. A large part of the metabolisable energy in forage maize is derived from starch in the kernel. The fibre‐rich remainder of the plant (the stover) also contains a significant amount of metabolisable energy and nutrients, despite its lower degradability. However, research over the past decades has mainly focused on improving yield and proportion of starch in forage maize in order to obtain a greater nutritional value and accommodate more to the nutritional needs of cattle.^1^ This focus did not lead to greater and/or more rapid degradation of the non‐starch fraction of the plant (mainly cell walls).^1^ Research into the causes of differences in degradability of the cell walls may result in better and faster degradability of cell walls of maize stems. Increased cell wall degradation of forage maize by harvesting at earlier stages of maturity will decrease starch content and increase enteric methane production.^2^ However, forage maize with a greater cell wall degradation at a similar growth stage will have a greater nutritional value, so lower costs, lower environmental emissions and a better performance of the animals.

The nutritive value of maize stover can vary widely and be affected by genotype, climate, maturity of the plant, and so on.^3^ The relationship between the degradability of maize stem cell walls and the different properties of the plant is well documented. For example, there is a strong negative correlation between cell wall maturation and degradability, which generally is ascribed to the increasing amount of acid detergent lignin (ADL) compared with other cell wall compounds.^4^ However, ADL content cannot fully explain the variation in cell wall degradability, with evidence that the ADL content may differ between cultivars without differences in cell wall degradability.^5^

Lignin is an organic polymer made up of phenyl propane units organised in a three‐dimensional structure. The precursors of these building blocks are coniferyl, sinapyl and p‐coumaryl alcohols, which can be transformed into guaiacyl (G unit), syringyl (S unit) and p‐hydroxyphenyl (H unit) units respectively through a complex dehydrogenative polymerisation process.^6^ Lignin content and composition change during plant maturation when more lignified primary and secondary cell walls of sclerenchyma and vascular tissues are developed.^7^ Studies on forages of different physiological maturation indicate a shift towards a more S‐unit‐type lignin with advancing maturity in some species.^8^, ^9^, ^10^ As already mentioned, maturation with an increasing content of ADL reduces forage degradability. In view of the complexity of factors involved in cell wall development, insufficient information exists on which factors (ADL content or composition) precisely determine the degradability of maize stem cell walls.

This study focuses on the relationship between the chemical and molecular properties of maize stem cell walls and in vitro rumen fermentation, measured by an automated gas production technique. The goal is to provide further insights into the background of differences in the composition of cell walls among different samples, at a chemical and molecular level, and the relationship with degradability.

## MATERIALS AND METHODS

### Maize production and management

Seeds of the maize cultivars Ambrosini and Aastar (provide by Limagrain, Rilland, the Netherlands) were sown in the first week of May 2012 at the experimental fields of Unifarm in Wageningen, the Netherlands. The plants' sowing density was 10 m^−2^, with 13.3 cm between plants and 75 cm between the rows. The fields had a sandy soil with pH 5.5, 21 g kg^−1^ organic matter (OM) and adequate levels of macro‐ and micronutrients. The fields were fertilized with 40 kg ha^−1^ of cow manure, 150 kg ha^−1^ of calcium ammonium nitrate (CAN, 270 g kg^−1^ N), 160 kg ha^−1^ of potassium (K60, 600 g kg^−1^ K_2_O) and 100 kg ha^−1^ phosphate (triple superphosphate, 450 g kg^−1^ P_2_O_5_).

### Sample preparation

During 2012, the internodes were harvested excluding the lower and upper nodes. Only internode 7 (counted from the ground) was harvested on 28 and 14 days before anthesis (d −28 and d −14 respectively) and on 14, 28, 42 and 70 days after anthesis (d 14, d 28, d 42 and d 70 respectively). As the major changes occurred after anthesis and levelled off afterwards, the interval between the last two sampling dates was 4 weeks instead of 2 weeks. At anthesis (14 August), whole plants were harvested and internodes 5, 7, 9, 11, 13 and 15 were collected. In all cases, internodes were collected from 12 plants (four plots, three plants per plot) and randomly separated to be duplicated. All the internodes were stored at −20 °C directly after harvesting.

All the internodes were oven‐dried at 70 °C and ground to pass a 1 mm sieve using a Peppink 100 AN cross‐beater mill (Peppink, Deventer, the Netherlands) before chemical analysis, pyrolysis gas chromatography–mass spectrometry (Py‐GC–MS) analysis, and in vitro fermentation.

### Chemical analysis

Dry matter (DM) was determined gravimetrically after 4 h at 103 °C, and ash after 3 h at 550 °C. Neutral detergent fibre (NDF) was determined by the method of Van Soest et al.,^11^ using a heat‐resistant amylase and expressed exclusive of residual ash. Acid detergent fibre (ADF) and ADL were determined by the method of Van Soest and McQueen^12^ and also expressed exclusive of residual ash. The difference between NDF and ADL was defined as potentially rumen degradable fibre (pRDF). Nitrogen (N) was determined by the Kjeldahl method, and crude protein was calculated as N × 6.25.

### Pyrolysis gas chromatography–mass spectrometry analysis

Pyrolysis of 100 µg, weighed on a Mettler‐Toledo XP6 microbalance (Mettler‐Toledo, Columbus, USA) was performed with an EGA/PY‐3030D micro‐furnace pyrolyser (Frontier Laboratories, Fukushima, Japan) connected to a Thermo7820A gas chromatograph using a DB‐1701 fused‐silica capillary column (60 m × 0.25 mm internal diameter, 0.25 µm film thickness) coupled to a DSQ‐II thermo mass‐selective detector (electron impact at 70 eV) (Thermo Scientific, Waltham, MA, USA). The pyrolysis was performed at 500 °C. The oven temperature was programmed from 45 °C (0–4 min) to 280 °C (5–60 min) at 4 °C min^−1^. Helium was the carrier gas (1 mL min^−1^). Species coming from lignin units and species coming from p‐coumaric and ferulic acids were distinguished assuming more than 80–85% of p‐coumaric and ferulic acids was considered part of lignin and not xylan, also based on the ratios of esterified p‐coumaric and ferulic acids to xylan reported by Van Dongen et al. 13 The compounds were identified by comparing their mass spectra with those of the Wiley and NIST libraries and with those reported in literature.^14^ The relative abundance of each identified compound was calculated based on the total relative area obtained from the pyrogram according to Jurak et al. 15 All the compounds identified and their spectra were checked manually. As the method is time consuming, not all samples were analysed, but enough were to see differences and trends. One sample from the duplicated samples of internodes 5, 9, 13 and 15 of both maize cultivars harvested at anthesis, and one sample from duplicated samples of internode 7 of both maize cultivars harvested on d −28, d 0, d 28 and d 70 were analysed using Py‐GC–MS. The syringyl:guaiacyl (S:G) ratio was calculated by dividing the sum of the abundance of the syringyl compounds by the sum of the abundance of the guaiacyl compounds.

### In vitro gas production

The fermentation kinetics were determined with the in vitro gas production technique.^16^ Incubation of 0.5 g of OM was performed in 60 mL buffered rumen fluid (one part of rumen fluid and two parts of buffer) in 250 mL bottles at 39 °C in a shaking water bath. Each sample was run in one bottle each time and each sample was run twice. Gas production was recorded for 72 h with an automated system.^16^ Results were corrected for blank gas productions; that is, buffered rumen fluid but without a substrate.

Rumen fluid was obtained from two non‐lactating cows that were fed twice a day with hay, and with 1 kg of concentrate in the morning. Rumen fluid was collected 2 h after the morning feeding and was pooled, stored in a warm insulated flask filled with CO_2_, filtered through cheesecloth, and mixed with an anaerobic buffer/mineral solution as described by Cone et al. 16 All processing of rumen fluid took place under continuous flushing with CO_2_.

The three‐phasic mathematical model for gas production as described by Cone et al. 16 and Groot et al. 17 was used to determine OM and cell wall degradation. The gas production curves are divided into three different sub‐curves, each with an asymptote (A), a half‐time value (B) and a shape parameter (C).^17^ The asymptotes of sub‐curve 1 (A1) correspond to the gas production between 0 and 3 h incubation and are caused by fermentation of the water‐soluble components, and those of sub‐curve 2 (A2) correspond to the gas production between 3 and 20 h incubation caused by fermentation of the non‐soluble components.^18^ The half‐time value B is the incubation time (hours) needed to reach half of the maximum gas production, representing a measure for the rate of degradation of the total OM.

### Statistical analysis

Data were analysed using the GLM procedure of SAS/STAT^®^ 9.3 (Statistical Analysis System, Cary, NC, USA) and the model included maize cultivar (Ambrosini and Aastar), maturity (different harvest dates or different internodes), and cultivar × maturity as fixed effects. Because Py‐GC–MS analyses were performed on one sample of selected internodes and harvest dates only, no interaction effect was included in the model when analysing S:G ratio data. Differences among main effects were analysed using Tukey–Kramer's multiple comparison procedure in the LSMEANS statement of SAS. Significant effects were declared at P ≤ 0.05 and trends at 0.05 < P ≤ 0.10.

## RESULTS

### Chemical composition of internode 7 at different harvest dates

The levels of ash, NDF, ADF, ADL, the ratio of lignin to pRDF (ADL:pRDF ratio) and the S:G ratio in internode 7, harvested at different dates during 2012, are shown in Table 1. Cultivar, harvest date and their interactions showed significant effects on the concentrations of ash, NDF, ADF and ADL and on the ADL:pRDF ratio. There was no clear trend for a greater level of NDF, ADF and ADL in more mature tissue. The ADL:pRDF ratio increased to the highest value on d 28 in Ambrosini and d 14 in Aastar and then decreased in both cultivars. The concentrations of NDF, ADF, and ADL and ADL:pRDF ratio were greater in Ambrosini than in Aastar except for the internode harvested on d 14. Cultivar and harvest date also showed significant effects on the S:G ratio, with a greater S:G ratio observed in Ambrosini than in Aastar and the lowest value of the S:G ratio observed in the youngest internode harvested on d −28 for both cultivars.

**Table 1:** Ash content (g kg^−1^ DM ± sd), NDF, ADF and ADL content (g kg^−1^ OM ± sd), ADL:pRDF ratio, S:G ratio and in vitro gas production (mL g^−1^ OM ± sd) parameters of fermentation of internode 7 of two maize cultivars (Ambrosini and Aastar) harvested on different dates (days before anthesis expressed with minus sign) in 2012

Cultivar	Harvest date	Ash	NDF	ADF	ADL	ADL:pRDF	S:G	A1	A2	GP72	B
Ambrosini	d −28	71 ± 0.9^a^	715 ± 6.4^c*^	487 ± 5.3^c*^	51 ± 0.1^d*^	7.73 ± 0.06^b*^	0.62	43 ± 1.2^cd^	117 ± 2.9^a*^	249 ± 6.3^bc*^	12.9 ± 0.5^d^
	d −14	47 ± 0.4^d^	739 ± 2.5^b*^	515 ± 8.2^b^	69 ± 4.4^bc*^	10.25 ± 0.80^ab*^	ND	39 ± 1.1^d^	91 ± 3.8^cd*^	233 ± 8.0^cd*^	16.8 ± 1.1^b^
	Anthesis	40 ± 0.2^e*^	598 ± 1.1^d*^	421 ± 0.6^d*^	59 ± 0.0^cd*^	10.92 ± 0.01^a*^	0.72	66 ± 3.4^a*^	95 ± 3.1^c*^	258 ± 12.4^ab^	11.8 ± 0.4^de^
	d 14	41 ± 0.1^e*^	589 ± 2.6^d*^	414 ± 0.2^d*^	50 ± 4.1^d^	9.36 ± 0.92^ab^	ND	72 ± 3.9^a*^	106 ± 2.1^b*^	273 ± 5.9^a*^	10.4 ± 0.3^e*^
	d 28	51 ± 0.6^c*^	719 ± 8.2^c*^	515 ± 9.1^b*^	78 ± 7.9^ab*^	12.27 ± 1.63^a^	0.83	47 ± 4.7^bc*^	95 ± 5.0^c*^	245 ± 11.9^bcd*^	14.9 ± 0.7^c*^
	d 42	58 ± 0.3^b*^	796 ± 1.4^a*^	565 ± 0.3^a*^	86 ± 1.7^a*^	12.12 ± 0.30^a*^	ND	30 ± 3.4^e*^	85 ± 5.6^d*^	227 ± 11.4^d*^	19.8 ± 0.5^a*^
	d 70	58 ± 0.0^b*^	705 ± 3.5^c*^	489 ± 1.9^c*^	65 ± 1.8^bcd*^	10.16 ± 0.28^ab^	0.79	54 ± 2.6^b*^	86 ± 3.7^cd*^	246 ± 5.0^bcd*^	15.8 ± 0.4^bc*^
Aastar	d −28	69 ± 0.5^b^	672 ± 3.7^bc^	448 ± 0.2^b^	34 ± 0.6^c^	5.34 ± 0.08^c^	0.49	41 ± 0.7^e^	131 ± 4.0^a^	264 ± 5.0^bd^	13.4 ± 0.1^b^
	d −14	48 ± 0.7^d^	715 ± 2.2^a^	490 ± 0.9^a^	49 ± 0.3^b^	7.42 ± 0.02^bc^	ND	38 ± 3.1^e^	118 ± 3.5^ac^	267 ± 8.9^cde^	16.3 ± 0.5^a^
	Anthesis	41 ± 0.0^f^	554 ± 0.0^e^	376 ± 2.8^d^	42^bc^	8.25^ab^	0.61	77 ± 3.6^ab^	109 ± 10.7^cd^	284 ± 23.2^abc^	11.2 ± 0.6^cd^
	d 14	50 ± 0.8^c^	678 ± 2.6^b^	480 ± 1.3^a^	66 ± 4.6^a^	10.72 ± 0.83^a^	ND	54 ± 5.0^d^	102 ± 2.5^d^	258 ± 7.7^cd^	13.2 ± 0.7^b^
	d 28	44 ± 0.4^e^	552 ± 7.9^e^	375 ± 1.3^d^	42 ± 0.7^bc^	8.27 ± 0.28^b^	0.65	83 ± 5.1^a^	124 ± 4.1^ab^	310 ± 11.6^a^	10.4 ± 0.4^d^
	d 42	50 ± 0.1^c^	586 ± 3.0^d^	404 ± 3.1^c^	46 ± 1.4^bc^	8.44 ± 0.24^ab^	ND	73 ± 1.7^bc^	113 ± 6.8^bcd^	296 ± 20.7^abe^	12.6 ± 0.9^bc^
	d 70	71 ± 0.2^a^	660 ± 4.1^c^	441 ± 10.4^b^	44 ± 5.3^bc^	7.14 ± 1.05^bc^	0.65	65 ± 3.4^c^	114 ± 4.3^bcd^	282 ± 7.2^ad^	12.9 ± 0.8^b^
Significance P	Cultivar C	<0.001	<0.001	<0.001	<0.001	<0.001	0.003	<0.001	<0.001	<0.001	<0.001
	Date D	<0.001	<0.001	<0.001	<0.001	<0.001	0.009	<0.001	<0.001	0.001	<0.001
	C × D	<0.001	<0.001	<0.001	<0.001	0.002	—	<0.001	<0.001	<0.001	<0.001

DM, dry matter; sd, standard deviation; NDF, neutral detergent fibre; ADF, acid detergent fibre; ADL, acid detergent lignin; OM, organic matter; ADL:pRDF ratio (the ratio of ADL and potentially rumen degradable fibre (pRDF; calculated as the difference between NDF and ADL)) × 100; S:G ratio, the ratio of syringyl and guaiacyl compounds; A1 and A2, gas production within 3 h and between 3 and 20 h; B, time needed to reach half of GP72; GP72, gas production within 72 h. Values with different superscript letters (a, b, c, d, e) within cultivar are significantly different. Values with * are significantly (P ≤ 0.05) different from corresponding harvest dates of Aastar.

### Chemical composition of several internodes harvested at anthesis

The levels of ash, NDF, ADF, ADL, ADL:pRDF ratio and S:G ratio in successive internodes within the stem harvested at anthesis for the two cultivars are shown in Table 2. Cultivar and internode significantly affected all these parameters and a significant cultivar × internode effect for ash, NDF, ADF and ADL:pRDF ratio was found. Again, no clear trend for a greater level of NDF in both cultivars and ADF in Ambrosini in older internodes (lower internode number) was observed. However, there was a clear trend for a lower concentration of ADL and a lower ADL:pRDF ratio and S:G ratio in younger tissues (higher internodes) for both cultivars. Aastar had significantly lower concentrations of NDF, ADF and ADL and a lower ADL:pRDF ratio and S:G ratio than Ambrosini.

**Table 2:** Ash content (g kg^−1^ DM ± sd), NDF, ADF and ADL content (g kg^−1^ OM ± sd), ADL:pRDF ratio, S:G ratio and in vitro gas production (mL g^−1^ OM ± sd) parameters of fermentation of internodes (5, 7, 9, 11, 13 and 15) of two maize cultivars (Ambrosini and Aastar) harvested at anthesis in 2012

Cultivar	Internode	Ash	NDF	ADF	ADL	ADL:pRDF	S:G	A1	A2	GP72	B
Ambrosini	5	76 ± 0.1^a*^	739 ± 1.4^a*^	521 ± 0.8^a*^	78 ± 0.5^a*^	11.88 ± 0.06^a*^	0.90	39 ± 8.4^b*^	97 ± 6.3^c*^	245 ± 21.3^b*^	17.1 ± 1.4^a*^
	7	44 ± 0.4^b^	646 ± 0.6^e*^	445 ± 2.3^b*^	63 ± 2.2^b*^	10.73 ± 0.42^b*^	ND	58 ± 10.8^ab^	111 ± 4.6^bc*^	282 ± 16.0^ab*^	14.6 ± 1.2^ab*^
	9	32 ± 0.7^e*^	686 ± 2.5^b*^	455 ± 1.3^b*^	60 ± 0.5^bc*^	9.58 ± 0.13^c*^	0.66	50 ± 8.5^ab*^	112 ± 5.4^bc*^	266 ± 15.5^ab*^	14.2 ± 1.6^ab*^
	11	35 ± 0.6^d^	664 ± 0.7^d*^	418 ± 4.4^c*^	57 ± 0.0^cd*^	9.41 ± 0.01^cd*^	ND	60 ± 2.5^a^	139 ± 17.0^a^	313 ± 44.0^a^	12.5 ± 1.9^b^
	13	37 ± 0.1^c^	671 ± 0.0^c*^	403 ± 1.4^d*^	51 ± 1.0^e*^	8.25 ± 0.17^e*^	0.47	60 ± 8.2^a^	132 ± 9.9^a^	299 ± 26.4^ab^	12.2 ± 1.6^b^
	15	36 ± 0.4^cd*^	674 ± 2.1^c^	407 ± 4.2^cd*^	53 ± 1.6^de*^	8.53 ± 0.27^de*^	0.53	51 ± 3.0^ab^	123 ± 2.7^ab*^	274 ± 9.1^ab^	13.0 ± 0.1^ab^
Aastar	5	60 ± 0.3^a^	599 ± 1.3^b^	421 ± 4.0^a^	57 ± 0.1^a^	10.51 ± 0.01^a^	0.73	60 ± 3.3^bc^	120 ± 10.2^b^	291 ± 18.2	13.6 ± 0.7^a^
	7	45 ± 0.1^b^	562 ± 2.8^de^	388 ± 0.1^b^	43 ± 1.2^b^	8.38 ± 0.20^b^	ND	71 ± 4.6^ab^	133 ± 6.7^ab^	323 ± 15.0	12.7 ± 0.4^ab^
	9	36 ± 0.1^e^	555 ± 2.3^e^	359 ± 1.8^c^	38 ± 1.6^c^	7.32 ± 0.30^c^	0.53	74 ± 4.4^a^	137 ± 2.7^ab^	327 ± 8.0	11.7 ± 0.4^b^
	11	34 ± 0.0^f^	577 ± 2.4^cd^	356 ± 2.3^c^	38 ± 0.0^c^	6.89 ± 0.10^cd^	ND	67 ± 5.9^ac^	139 ± 4.7^ab^	316 ± 16.6	11.7 ± 0.3^b^
	13	38 ± 0.3^d^	589 ± 10.8^bc^	350 ± 7.1^c^	33 ± 0.8^cd^	6.01 ± 0.27^de^	0.45	62 ± 7.1^ac^	142 ± 10.4^a^	310 ± 25.8	11.5 ± 0.9^b^
	15	41 ± 0.3^c^	659 ± 5.9^a^	375 ± 1.1^b^	32 ± 2.2^d^	5.11 ± 0.42^e^	0.39	55 ± 6.4^c^	146 ± 8.0^a^	306 ± 27.4	11.8 ± 0.9^b^
Significance P	Cultivar C	<0.001	<0.001	<0.001	<0.001	<0.001	0.039	<0.001	<0.001	<0.001	<0.001
	Internode I	<0.001	<0.001	<0.001	<0.001	<0.001	0.012	0.001	<0.001	0.010	<0.001
	C × I	<0.001	<0.001	<0.001	0.169	0.002	‐	0.018	0.081	0.164	0.191

DM, dry matter; sd, standard deviation; NDF, neutral detergent fibre; ADF, acid detergent fibre; ADL, acid detergent lignin; OM, organic matter; ADL:pRDF ratio (the ratio of ADL and potentially rumen degradable fibre (pRDF; calculated as the difference between NDF and ADL)) × 100; S:G ratio, the ratio of syringyl and guaiacyl compounds; A1 and A2, gas production within 3 h and between 3 and 20 h; B, time needed to reach half of GP72; GP72, gas production within 72 h. Values with different superscript letters (a, b, c, d, e) within cultivar are significantly different. Values with * are significantly (P ≤ 0.05) different from corresponding harvest dates of Aastar.

### In vitro fermentation of internode 7 at different harvest dates

The results of the kinetics of gas production of internode 7 harvested at different dates of the year are shown in Table 1. Cultivar, harvest date and the interactions showed significant effects on A1, A2, total gas production after 72 h (GP72) and B. For A1, B and GP72 for both Ambrosini and Aastar, there was no clear increase or decrease at later harvest dates. The earliest harvested internodes had the highest A2, and there was a tendency for lower gas production as the internodes were harvested later.

### In vitro fermentation of several different internodes harvested at anthesis

The results of the kinetics of gas production of internodes 5, 7, 9, 11, 13 and 15 harvested on the same day for the two cultivars are shown in Table 2. Cultivar and internode showed significant effects on A1, A2, GP72 and B. The interactions between cultivar and internode only influenced A1 significantly. There was no clear trend for A1. A greater A2 was observed when the internode was younger (higher internode) in the plant for Aastar, while the lowest internode (internode 5) showed the lowest A2 and GP72 for both cultivars. The half‐time value B tended to decrease from lower to higher internodes.

### Relationship between ADL content or S:G ratio and in vitro fermentation

**Figure 1:**
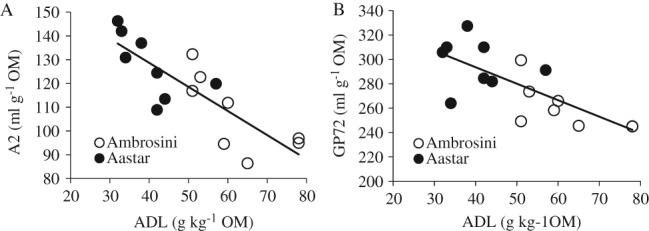
(A) Relationship between ADL content and in vitro gas production between 3 and 20 h incubation (parameter A2) of the internodes 5, 9, 13 and 15 harvested at anthesis and internode 7 harvested at 28 days before anthesis, at anthesis, and 28 and 70 days after anthesis from two maize cultivars (Ambrosini and Aastar): A2 = − 1.01 ± 0.19 × ADL + 169 ± 9.9 (estimate plus/minus standard error), root‐mean‐square error 10.54, R^2^ = 0.68. (B) Relationship between ADL content and in vitro gas production within 72 h incubation (GP72) of the internodes 5, 9, 13 and 15 harvested at anthesis and internode 7 harvested at 28 days before anthesis, at anthesis, and 28 and 70 days after anthesis from two maize cultivars (Ambrosini and Aastar): GP72 = − 1.35 ± 0.33 × ADL + 348 ± 17.6 (estimate plus/minus standard error), root‐mean‐square error 18.75, R^2^ = 0.54.

The relationships between ADL content and A2 and GP72 are shown in Fig. 1. The relationship between ADL content and A2 (indicating cell wall degradation) was stronger (R^2^ = 0.68) than the relationship between ADL content and GP72 (indicating OM degradation) (R^2^ = 0.54).

**Figure 2:**
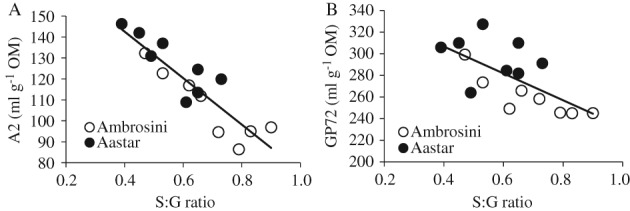
(A) Relationship between S:G ratio and in vitro gas production between 3 and 20 h incubation (parameter A2) of the internodes 5, 9, 13 and 15 harvested at anthesis and internode 7 harvested at 28 days before anthesis, at anthesis, and 28 and 70 days after anthesis from two maize cultivars (Ambrosini and Aastar), A2 = − 110.99 ± 14.85 × S:G ratio + 187 ± 9.5 (estimate plus/minus standard error), root‐mean‐square error 8.32, R^2^ = 0.80. (B) Relationship between S:G ratio and in vitro gas production within 72 h incubation (GP72) of the internodes 5, 9, 13 and 15 harvested at anthesis and internode 7 harvested at 28 days before anthesis, at anthesis, and 28 and 70 days after anthesis from two maize cultivars (Ambrosini and Aastar), GP72 = − 124.27 ± 36.67 × S:G ratio + 356 ± 23.5 (estimate plus/minus standard error), root‐mean‐square error 20.54, R^2^ = 0.45.

The relationships between S:G ratio and A2 and GP72 are shown in Fig. 2. The relationship between S:G ratio and A2 was stronger (*R*^2^ = 0.80) than the relationship between S:G ratio and GP72 (*R*^2^ = 0.45). Upon comparison with the relationships between ADL content and A2, the results indicate that the S:G ratio plays a more dominant role in cell wall degradation than the ADL content; however, in comparison with ADL content, the S:G ratio appeared not to have a strong relationship with OM degradation.

## DISCUSSION

### Effect of maturity on chemical composition

The developing internodes of maize stem provide the opportunity to study how cell wall composition changes during maturation.^19^, ^20^, ^21^ Different maturity stages can be represented by successive internodes of the maize stem harvested at the same time^19^ or by selecting a given internode and harvesting it at different dates.^21^ Within the stem, cell walls of the upper part (greater internode number) are physiologically younger and less lignified than those of the lower part. In this study, internodes 5, 7, 9, 11, 13 and 15 sampled on the same day and internode 7 sampled on different days during the growth were used to investigate how maturity influenced the chemical composition of cell walls, as well as cell wall degradation.

Cone and Engels^5^ reported that NDF and ADF increased with increasing maturity, which was also demonstrated by Tolera and Sundstøl.^22^ However, NDF and ADF did not have the clear increasing trend with maturity in the present study, which is in line with the results reported by Boon *et al*.^3^ and Cone *et al*.^23^ In contrast to expected changes in the ADL content of the stems,^5^, ^22^ in this study the ADL content did not increase when harvest date was used as an indicator of maturity. When different internodes sampled at anthesis were used to investigate the changes in the ADL content, the ADL content, as expected, increased from the upper internodes (internode 15, average 43 g kg^−1^ OM) to the lower internodes (internode 5, average 68 g kg^−1^ OM). The ADL:pRDF ratio, as an indication of potentially degradable OM, is expected to be greater in older internodes, which is demonstrated by different internodes harvested on the same date. However, when internode 7 was harvested on different days, an increasing ADL:pRDF ratio in early growth was observed followed by a trend to decrease in later growth.

The S:G ratio increased during the maturation, especially from the top to the bottom of the stem. This observation is in line with data from other studies.^6^, ^8^, ^24^ Deposition of G units continues throughout the lignification of cell walls, while large amounts of S units are deposited mainly in the middle and late stages of lignification.^6^, ^25^ Therefore, the composition of lignin shifts from lignin with primarily G units to lignin with mixed S–G units during cell wall development and the S:G ratio increases.

### Effect of maturity on cell wall degradation

In this study, the *in vitro* gas production technique was used to assess the cell wall degradation. In the case of maize stems, cell wall degradability is represented by A2, being the gas production caused by fermentation of the non‐soluble fraction, while OM degradability is represented by GP72 (the gas produced after 72 h fermentation) as indicated by Cone and co‐workers^16^, ^18^ and Groot *et al*.^17^

The decline in cell wall degradability during maturation is well documented, and it is generally accepted that the formation of more lignified plant cell walls during maturation is the major reason leading to lower cell wall degradation.^26^ Jung and Casler^27^ found that the degradability of cell walls in maize stems after both 24‐ and 96‐h *in vitro* incubations with rumen fluid decreased as the stems were harvested later. Our results show that there was a clear trend that the cell wall degradability decreased only from the younger internode to the older internode within the stem. For internode 7, sampled on different dates, there was rapid drop in cell wall degradability up to 14 August, to remain fairly constant afterwards, which is in line with Cone and Engels,^5^ who reported that the cell wall degradability decreased significantly up to 15 August and remained fairly constant afterwards when the date of sowing was 25 April, which is close to the date of sowing in our study.

### Factors related to cell wall degradability

Lignin content of forages has long been reported to be negatively correlated with cell wall degradability,^28^ and this relationship, which was also observed here, is consistent with previous studies.^29^, ^30^, ^31^, ^32^, ^33^ Boon *et al*. found that the lignin content in the internode 7 of corn stem was significantly correlated with cell wall degradation assessed by *in vitro* gas production.^29^ The negative correlation between lignin content and cell wall degradation was also reported in both grass^30^, ^31^, ^32^ and legumes.^31^, ^33^ Even though these studies demonstrated that lignin content is an important factor that limits cell wall degradation in ruminants, the strength of the relationship varied among studies due to the methods that were used to evaluate cell wall degradation. However, comparing different corn genotypes, Cone and Engels^5^ suggested that ADL content is not always a good indicator of degree of cell wall degradability, which is supported by Sommerfeldt *et al*.,^34^ who observed that degradability of cell walls was greater for bm3 maize than for a normal variety although no differences in ADL content were observed. However, both a reduced ADL content and a lower S:G ratio were found in bm3 maize in other studies.^35^, ^36^ The relationship between ADL content or ADL composition and cell wall degradability is not fully understood even though this relationship has been investigated for many years. Compared with the S:G ratio, the ADL content has a weaker relationship with cell wall degradability (Figs 1A and 2A). This result is in accordance with what Vailhe *et al*.^37^ and Sewalt *et al*.^38^ observed for tobacco. Guo *et al*.^39^ found that a greater S:G ratio in alfalfa resulted in a greater cell wall digestibility, which is the opposite of our results. The significant correlation between the S:G ratio and the cell wall degradability in maize stems was not found by Jung and Buxton^40^ and Grabber *et al*..^41^ It should be noticed that only a single maturity stage was used in the research conducted by Jung and Buxton^40^ and by Grabber *et al*.,^41^ which may be the reason of the discrepancy between the results of these studies. When forages were harvested across maturity stages, strong negative correlations between ADL content and cell wall degradability were shown,^42^, ^43^ while only weak negative correlations existed between ADL content and cell wall degradability if forages of a single maturity stage were examined.^40^, ^44^

The reason why a lower S:G ratio is related to greater degradability is difficult to explain. Jung and Deetz^26^ hypothesised that syringyl‐rich lignin would be more inhibitory to cell wall degradation, as syringyl monolignols have fewer potential polymerisation sites and should have a more linear polymer structure which could protect a larger area of the secondary cell walls from degradation than the more branched guaiacyl‐rich lignin. However, Filley *et al*.^45^ emphasized that the G‐type lignin is more resistant to chemical and biological breakdown than the S‐type lignin is. In all likelihood, the influence of the S:G ratio on cell wall degradability should not be explained by the nature of the S or G unit itself in view of the complex linkages between S or G units with other molecular structures that may be related to a reduction in degradability. One possible explanation is that, during the formation of S units, mostly *p*‐coumaric acid is connected with the S unit through ester bonds^46^ and *p*‐coumaric acid is thought to be more toxic to ruminal microorganisms than other phenolic acids and may limit cell wall degradation.^47^ The latter is supported by Martínez *et al*.,^48^ who reported that removal of *p*‐coumarate in sugar cane bagasse enhanced the enzyme degradation of cellulose and led to a greater cell wall degradation.

## CONCLUSIONS

The ADL content and the S:G ratio increased from the upper internode to the lower internode within the maize stem for both cultivars investigated (Aastar and Ambrosini), and the S:G ratio in internode 7 tended to increase during the growing season. However, the ADL content in internode 7 fluctuated during maturation. Cell wall degradability, as determined with the gas production technique, tended to decrease up to anthesis with no clear pattern after anthesis, whereas cell wall degradability increased with internode number (from bottom to top).

For maize stems, the S:G ratio (lignin composition) showed a better relationship with cell wall degradability than the ADL content. A lower S:G ratio was associated with a greater degradability.

## Supporting information

Appendix S1. Supporting information
