# Supplementary material for: Lignin composition is more important than content for maize stem cell wall degradation
Source: J Sci Food Agric. 2017 Sep 25;98(1):384–90. doi: 10.1002/jsfa.8630 (PMC5725715; doi:10.1002/jsfa.8630)
Supplement: Supplementary file 1 — Appendix S1. Supporting information [file JSFA-98-384-s001.docx]

Below is all data from pyrolysis gas chromatography / mass spectrometry analysis of the samples. The structures of the components are published by Jurak *et al.*^1^ and by Martínez *et al.*.^2^

|  | Compound | Origin^a^ | Ambrosini | | | | Aastar | | | |
| --- | --- | --- | --- | --- | --- | --- | --- | --- | --- | --- |
|  |  |  | 17  jul | 14  aug | 11  sep | 23  oct | 17  jul | 14  aug | 11  sep | 23  oct |
| 1 | Furfural | C | 8.3 | 7.9 | 5.8 | 6.9 | 9.1 | 7.2 | 7.1 | 7.5 |
| 2 | (5H)-furan-2-one | C | 3.8 | 3.5 | 3.5 | 2.9 | 4.4 | 3.8 | 3.9 | 3.1 |
| 3 | 2-acetylfuran | C | 1.0 | 1.0 | 0.8 | 0.8 | 1.3 | 1.0 | 0.9 | 1.0 |
| 4 | 4-hydroxy-5,6-dihydro-2H-pyran-2-one | C | 0.7 | 1.2 | 0.5 | 0.6 | 0.8 | 1.5 | 1.2 | 0.5 |
| 5 | 1,4-anhydroarabinofuranose | C | 0.1 | 0.1 | 0.0 | 0.1 | 0.0 | 0.1 | 0.1 | 0.0 |
| 6 | 5-(hydroxymethyl)dihydro-2(3H)-furanone | C | 0.9 | 1.2 | 0.9 | 0.8 | 1.2 | 1.7 | 1.3 | 0.9 |
| 7 | 5-hydroxymethylfurfural | C | 0.1 | 1.8 | 0.0 | 0.6 | 0.3 | 1.9 | 1.2 | 1.1 |
| 8 | 2-methylfuran | C | 0.5 | 0.5 | 0.3 | 0.3 | 0.6 | 0.5 | 0.5 | 0.5 |
| 9 | 2,3-dihydro-5-methylfuran | C | 13.7 | 11.8 | 10.0 | 9.1 | 15.6 | 10.4 | 10.5 | 9.9 |
| 10 | 1,6-anhydro-β-D-glucopyranose (levoglucosan) | C | 0.4 | 1.4 | 0.1 | 0.5 | 0.4 | 0.7 | 0.8 | 0.4 |
|  |  |  |  |  |  |  |  |  |  |  |
| 11 | Phenol | H | 5.8 | 4.4 | 5.5 | 5.3 | 5.5 | 4.4 | 4.9 | 5.9 |
| 12 | 2-methylphenol (o-cresol) | H | 0.8 | 0.7 | 0.7 | 0.6 | 0.8 | 0.7 | 0.7 | 0.8 |
| 13 | 4-methylphenol (p-cresol) | H | 0.8 | 0.7 | 0.7 | 0.6 | 0.8 | 0.7 | 0.7 | 0.8 |
| 14 | 4-ethylphenol | H | 0.2 | 0.2 | 0.2 | 0.2 | 0.2 | 0.2 | 0.2 | 0.2 |
| 15 | 4-vinylphenol | H/ PCA | 38.7 | 41.8 | 42.7 | 42.9 | 39.1 | 42.9 | 43.2 | 42.7 |
| 16 | Hydroquinone | H | 0.7 | 0.7 | 0.5 | 0.7 | 0.9 | 0.9 | 0.8 | 0.9 |
|  |  |  |  |  |  |  |  |  |  |  |
| 17 | Guaiacol | G | 4.8 | 3.5 | 4.8 | 4.7 | 4.3 | 3.8 | 4.3 | 4.8 |
| 18 | 4-methylguaiacol | G | 0.3 | 0.5 | 0.4 | 0.5 | 0.3 | 0.5 | 0.5 | 0.4 |
| 19 | 4-ethylguaiacol | G | 0.5 | 0.7 | 0.6 | 0.6 | 0.5 | 0.6 | 0.7 | 0.7 |
| 20 | 4-vinylguaiacol | G/ FA | 7.3 | 5.7 | 7.4 | 7.1 | 6.3 | 6.3 | 5.9 | 6.5 |
| 21 | Eugenol | G | 0.0 | 0.1 | 0.1 | 0.1 | 0.0 | 0.1 | 0.1 | 0.1 |
| 22 | 4-propylguaiacol | G | 0.0 | 0.0 | 0.1 | 0.0 | 0.0 | 0.0 | 0.0 | 0.0 |
| 23 | Vanillin | G | 0.7 | 0.6 | 0.6 | 0.7 | 0.6 | 0.6 | 0.6 | 0.6 |
| 24 | *cis*-isoeugenol | G | 0.0 | 0.0 | 0.0 | 0.1 | 0.0 | 0.1 | 0.1 | 0.1 |
| 25 | *trans*-isoeuganol | G | 0.4 | 0.6 | 0.6 | 0.6 | 0.4 | 0.6 | 0.5 | 0.5 |
| 26 | Acetovanillone | G | 0.0 | 0.1 | 0.1 | 0.2 | 0.0 | 0.0 | 0.1 | 0.0 |
| 27 | Guaiacylacetone | G | 0.4 | 0.4 | 0.5 | 0.5 | 0.3 | 0.5 | 0.4 | 0.6 |
| 28 | *trans*-coniferaldehyde | G | 0.0 | 0.1 | 0.1 | 0.2 | 0.0 | 0.1 | 0.1 | 0.1 |
|  |  |  |  |  |  |  |  |  |  |  |
| 29 | Syringol | S | 5.3 | 5.0 | 6.9 | 6.4 | 3.7 | 4.4 | 5.0 | 5.4 |
| 30 | 4-methylsyringol | S | 0.4 | 0.4 | 0.5 | 0.5 | 0.3 | 0.4 | 0.5 | 0.4 |
| 31 | 4-vinylsyringol | S | 1.7 | 1.4 | 2.5 | 2.2 | 1.2 | 1.3 | 1.3 | 1.5 |
| 32 | 4-allyl-2,6-dimethoxyphenol | S | 0.0 | 0.2 | 0.2 | 0.2 | 0.0 | 0.1 | 0.1 | 0.1 |
| 33 | Syringaldehyde | S | 0.2 | 0.3 | 0.3 | 0.3 | 0.2 | 0.3 | 0.3 | 0.3 |
| 34 | *cis*-2,6-dimethoxy-4-propenylphenol | S | 0.1 | 0.1 | 0.2 | 0.1 | 0.0 | 0.1 | 0.1 | 0.1 |
| 35 | Homosyringaldehyde | S | 0.0 | 0.0 | 0.2 | 0.2 | 0.0 | 0.1 | 0.1 | 0.1 |
| 36 | *trans*-2,6-dimethoxy-4-propenylphenol | S | 0.6 | 0.7 | 1.0 | 1.0 | 0.5 | 0.7 | 0.7 | 0.8 |
| 37 | Acetosyringone | S | 0.3 | 0.3 | 0.3 | 0.4 | 0.2 | 0.3 | 0.3 | 0.3 |
| 38 | Syringylacetone | S | 0.3 | 0.3 | 0.4 | 0.4 | 0.2 | 0.3 | 0.3 | 0.4 |
| 39 | *trans*-sinapyl alcohol | S | 0.1 | 0.1 | 0.1 | 0.2 | 0.0 | 0.1 | 0.1 | 0.1 |
| Based on Pyrolysis GC-MS | % Carbohydrates^b^ | | 29.4 | 30.5 | 21.9 | 22.6 | 33.5 | 29.0 | 27.6 | 24.9 |
|  | % Lignin | | 70.6 | 69.5 | 78.1 | 77.4 | 66.5 | 71.0 | 72.4 | 75.1 |
|  | Ratio Lignin/Carbohydrate | | 2.40 | 2.28 | 3.56 | 3.42 | 1.98 | 2.45 | 2.62 | 3.02 |
|  | SUM H^c^ | | 46.9 | 48.4 | 50.2 | 50.2 | 47.3 | 49.7 | 50.3 | 51.2 |
|  | SUM G^c^ | | 14.6 | 12.3 | 15.2 | 15.2 | 12.8 | 13.2 | 13.4 | 14.5 |
|  | SUM S^c^ | | 9.1 | 8.9 | 12.7 | 12.0 | 6.3 | 8.0 | 8.7 | 9.4 |
|  | Ratio Syringyl/Guaiacyl | | 0.62 | 0.72 | 0.83 | 0.79 | 0.49 | 0.61 | 0.65 | 0.65 |
|  | Ratio (Syringyl/Guaiacyl)_except vinylguaiacol_^d^ | | 1.01 | 1.13 | 1.30 | 1.21 | 0.79 | 0.97 | 1.00 | 0.99 |
|  |  |  |  |  |  |  |  |  |  |  |
|  | % Cα-unsubstituted lignin | | 15.8 | 12.9 | 17.2 | 16.4 | 13.5 | 12.6 | 14.2 | 16.1 |
|  | % Cα-methylated lignin | | 2.2 | 2.2 | 2.3 | 2.2 | 2.3 | 2.3 | 2.3 | 2.3 |
|  | % Cα-vinyl lignin | | 47.7 | 48.8 | 52.5 | 52.1 | 46.6 | 50.5 | 50.4 | 50.8 |
|  | % Cα-oxidized lignin | | 2.0 | 2.0 | 2.2 | 2.5 | 1.6 | 1.9 | 1.9 | 2.2 |
|  | % Cα-oxidized G-units | | 1.2 | 1.1 | 1.2 | 1.3 | 0.9 | 1.1 | 1.1 | 1.3 |
|  | % Cα-oxidized S-units | | 0.9 | 0.9 | 1.0 | 1.2 | 0.6 | 0.8 | 0.9 | 0.9 |
| ^a^ C, carbohydrate-derived compound; H, *p*-hydroxycinnamyl lignin-derived compounds; G, guaiacyl lignin-derived compounds; S, syringyl lignin-derived compounds; PCA, *p*-coumarates; FA, ferulates. | | | | | | | | | | |
| ^b^ Standard deviations of the % of carbohydrates were < 2.0 | | | | | | | | | | |
| ^c^ Standard deviations of the sum of H, G and S compounds were < 1.5 | | | | | | | | | | |
| ^d^ All G and S derived peaks were used for the calculation of the S:G ratio except vinylguaiacol which also can arise from ferulates. | | | | | | | | | | |

|  | Compound | Origin^a^ | Ambrosini Internodes | | | | Aastar Internodes | | | |
| --- | --- | --- | --- | --- | --- | --- | --- | --- | --- | --- |
|  |  |  | 5 | 9 | 13 | 15 | 5 | 9 | 13 | 15 |
| 1 | Furfural | C | 8.4 | 8.5 | 10.5 | 9.7 | 7.5 | 8.2 | 9.4 | 10.6 |
| 2 | (5H)-furan-2-one | C | 3.0 | 4.3 | 4.5 | 4.9 | 3.6 | 4.3 | 5.7 | 5.6 |
| 3 | 2-acetylfuran | C | 0.8 | 0.7 | 1.0 | 1.0 | 0.7 | 0.8 | 1.0 | 1.1 |
| 4 | 4-hydroxy-5,6-dihydro-2H-pyran-2-one | C | 2.4 | 1.7 | 2.0 | 1.8 | 2.6 | 2.6 | 2.0 | 1.8 |
| 5 | 1,4-anhydroarabinofuranose | C | 0.2 | 0.1 | 0.3 | 0.2 | 0.1 | 0.1 | 0.2 | 0.2 |
| 6 | 5-(hydroxymethyl)dihydro-2(3H)furanone | C | 1.0 | 1.3 | 1.3 | 1.5 | 1.2 | 1.9 | 2.1 | 1.8 |
| 7 | 5-hydroxymethylfurfural | C | 1.3 | 0.6 | 0.7 | 0.3 | 2.4 | 1.5 | 0.7 | 0.4 |
| 8 | 2-methylfuran | C | 0.5 | 0.5 | 0.5 | 0.3 | 0.5 | 0.6 | 0.5 | 0.6 |
| 9 | 2,3-dihydro-5-methylfuran | C | 12.3 | 12.7 | 15.2 | 16.0 | 12.4 | 13.3 | 15.8 | 17.5 |
| 10 | 1,6-anhydro-β-D-glucopyranose (levoglucosan) | C | 1.8 | 0.5 | 1.4 | 0.7 | 0.8 | 0.8 | 0.4 | 0.4 |
|  |  |  |  |  |  |  |  |  |  |  |
| 11 | Phenol | H | 3.9 | 3.6 | 3.8 | 3.7 | 3.6 | 3.5 | 3.9 | 4.0 |
| 12 | 2-methylphenol (o-cresol) | H | 0.4 | 0.5 | 0.5 | 0.5 | 0.5 | 0.4 | 0.5 | 0.6 |
| 13 | 4-methylphenol (p-cresol) | H | 0.4 | 0.5 | 0.5 | 0.5 | 0.5 | 0.4 | 0.5 | 0.6 |
| 14 | 4-ethylphenol | H | 0.0 | 0.1 | 0.1 | 0.2 | 0.1 | 0.0 | 0.1 | 0.2 |
| 15 | 4-vinylphenol | H/PCA | 42.9 | 37.7 | 32.9 | 34.3 | 42.1 | 38.3 | 32.6 | 30.1 |
| 16 | Hydroquinone | H | 0.5 | 0.6 | 0.8 | 0.6 | 0.4 | 0.6 | 0.8 | 0.8 |
|  |  |  |  |  |  |  |  |  |  |  |
| 17 | Guaiacol | G | 3.1 | 4.4 | 4.6 | 4.4 | 3.4 | 4.0 | 4.6 | 4.7 |
| 18 | 4-methylguaiacol | G | 0.2 | 0.6 | 0.6 | 0.5 | 0.4 | 0.5 | 0.5 | 0.3 |
| 19 | 4-ethylguaiacol | G | 0.4 | 0.5 | 0.6 | 0.5 | 0.4 | 0.6 | 0.6 | 0.5 |
| 20 | 4-vinylguaiacol | G/ FA | 5.2 | 7.4 | 8.3 | 8.1 | 6.3 | 7.7 | 8.6 | 9.8 |
| 21 | Eugenol | G | 0.1 | 0.1 | 0.1 | 0.1 | 0.1 | 0.1 | 0.1 | 0.0 |
| 22 | 4-propylguaiacol | G | 0.0 | 0.0 | 0.0 | 0.0 | 0.0 | 0.0 | 0.0 | 0.0 |
| 23 | Vanillin | G | 0.6 | 0.7 | 0.8 | 0.7 | 0.5 | 0.7 | 0.7 | 0.7 |
| 24 | *cis*-isoeugenol | G | 0.0 | 0.1 | 0.0 | 0.1 | 0.1 | 0.1 | 0.0 | 0.0 |
| 25 | *trans*-isoeuganol | G | 0.4 | 0.9 | 0.6 | 0.4 | 0.4 | 0.6 | 0.6 | 0.4 |
| 26 | Acetovanillone | G | 0.2 | 0.1 | 0.1 | 0.1 | 0.0 | 0.1 | 0.0 | 0.0 |
| 27 | Guaiacylacetone | G | 0.3 | 0.5 | 0.4 | 0.5 | 0.4 | 0.3 | 0.5 | 0.5 |
| 28 | *trans*-coniferaldehyde | G | 0.2 | 0.2 | 0.2 | 0.1 | 0.1 | 0.1 | 0.1 | 0.1 |
|  |  |  |  |  |  |  |  |  |  |  |
| 29 | Syringol | S | 5.1 | 5.1 | 3.7 | 4.1 | 4.7 | 4.1 | 3.7 | 3.5 |
| 30 | 4-methylsyringol | S | 0.4 | 0.5 | 0.4 | 0.4 | 0.4 | 0.4 | 0.3 | 0.3 |
| 31 | 4-vinylsyringol | S | 1.6 | 2.2 | 1.5 | 1.9 | 1.5 | 1.2 | 1.5 | 1.5 |
| 32 | 4-allyl-2,6-dimethoxyphenol | S | 0.2 | 0.2 | 0.2 | 0.0 | 0.2 | 0.0 | 0.0 | 0.0 |
| 33 | Syringaldehyde | S | 0.4 | 0.3 | 0.2 | 0.2 | 0.4 | 0.3 | 0.2 | 0.2 |
| 34 | *cis*-2,6-dimethoxy-4-propenylphenol | S | 0.1 | 0.1 | 0.0 | 0.1 | 0.1 | 0.1 | 0.0 | 0.0 |
| 35 | Homosyringaldehyde | S | 0.1 | 0.2 | 0.2 | 0.2 | 0.2 | 0.2 | 0.2 | 0.1 |
| 36 | *trans*-2,6-dimethoxy-4-propenylphenol | S | 0.9 | 0.9 | 0.7 | 0.6 | 0.8 | 0.7 | 0.7 | 0.5 |
| 37 | Acetosyringone | S | 0.3 | 0.4 | 0.4 | 0.3 | 0.3 | 0.4 | 0.4 | 0.3 |
| 38 | Syringylacetone | S | 0.4 | 0.4 | 0.3 | 0.4 | 0.3 | 0.3 | 0.3 | 0.3 |
| 39 | *trans*-sinapyl alcohol | S | 0.1 | 0.1 | 0.1 | 0.1 | 0.1 | 0.1 | 0.1 | 0.1 |
| Based on Pyrolysis GC-MS | % Carbohydrates^b^ | | 31.6 | 30.9 | 37.3 ± 4.6 | 36.2 ± 2.2 | 31.9 | 34.1 | 37.8 | 40.0 |
|  | % Lignin | | 68.4 | 69.1 | 46.3 | 63.8 | 68.1 | 22.6 | 62.2 | 60.0 |
|  | Ratio Lignin/Carbohydrate | | 2.16 | 2.24 | 1.68 | 1.76 | 2.14 | 0.66 | 1.65 | 1.50 |
|  | SUM H^c^ | | 48.2 | 43.1 | 38.6 | 39.8 | 47.1 | 43.2 ± 2.2 | 38.5 | 36.1 |
|  | SUM G^c^ | | 10.6 | 15.7 | 16.3 ± 2.0 | 15.7 | 12.1 | 14.8 | 16.3 | 17.2 |
|  | SUM S^c^ | | 9.5 | 10.3 | 7.7 | 8.3 | 8.9 | 7.8 | 7.4 | 6.7 |
|  | Ratio Syringyl/Guaiacyl | | 0.90 | 0.66 | 0.47 | 0.53 | 0.73 | 0.53 | 0.45 | 0.39 |
|  | Ratio (Syringyl/Guaiacyl)_except vinylguaiacol_^d^ | | 1.47 | 0.99 | 0.76 | 0.85 | 1.28 | 0.93 | 0.77 | 0.71 |
|  |  |  |  |  |  |  |  |  |  |  |
|  | % Cα-unsubstituted lignin | | 12.1 | 13.1 | 12.1 | 12.3 | 11.6 | 11.6 | 12.1 | 12.2 |
|  | % Cα-methylated lignin | | 1.5 | 2.1 | 1.9 | 1.9 | 1.6 | 1.8 | 1.9 | 1.7 |
|  | % Cα-vinyl lignin | | 49.7 | 47.3 | 42.8 | 44.3 | 49.9 | 47.2 | 42.8 | 41.4 |
|  | % Cα-oxidized lignin | | 2.0 | 2.4 | 2.2 | 2.3 | 1.9 | 2.0 | 2.1 | 2.0 |
|  | % Cα-oxidized G-units | | 1.0 | 1.4 | 1.3 | 1.4 | 1.0 | 1.1 | 1.2 | 1.2 |
|  | % Cα-oxidized S-units | | 1.0 | 1.1 | 0.9 | 0.9 | 1.0 | 1.0 | 0.9 | 0.8 |
| ^a^ C, carbohydrate-derived compound; H, *p*-hydroxycinnamyl lignin-derived compounds; G, guaiacyl lignin-derived compounds; S, syringyl lignin-derived compounds; PCA, *p*-coumarates; FA, ferulates. | | | | | | | | | | |
| ^b^ Standard deviations of the % of carbohydrates were < 2.0 or indicated | | | | | | | | | | |
| ^c^ Standard deviations of the sum of H, G and S compounds were < 1.5 or indicated | | | | | | | | | | |
| ^d^ All G and S derived peaks were used for the calculation of the S:G ratio except vinylguaiacol which also can arise from ferulates. | | | | | | | | | | |

REFERENCES

1 Jurak E, Punt AM, Arts W, Kabel MA and Gruppen H, Fate of carbohydrates and lignin during composting and mycelium growth of *Agaricus bisporus* on wheat straw based compost. *PLoS One* **10.1371**:1-16 (2015).

2 Martínez PM, Punt AM, Kabel MA and Gruppen H, Deconstruction of lignin linked *p*-coumarates, ferulates and xylan by NaOH enhances the enzymatic conversion of glucan. *Bioresour Technol* **216**:44-51 (2016).
